# Effectiveness of Smartphone-Based Versus Community-Based Otago Exercise Programs for Fall Risk Reduction in Community-Dwelling Older Adults: Protocol for a Randomized Controlled Trial

**DOI:** 10.2196/102920

**Published:** 2026-11-05

**Authors:** Patima Silsupadol, Vipul Lugade, Woei-Nan Bair, Kelly Grier

**Affiliations:** 1 Division of Physical Therapy, Decker College of Nursing and Health Sciences, Binghamton University, 48 H. Stenger Place, Johnson City, NY, 13790, United States, 1 6077774846; 2 Motion Analysis Research Laboratory, Division of Physical Therapy, Decker College of Nursing and Health Sciences, Binghamton University, Binghamton, NY, United States

**Keywords:** otago exercise program, smartphone-based intervention, community-based program, fall prevention, older adults, mobile phone, digital health

## Abstract

**Background:**

The Otago Exercise Program (OEP), traditionally delivered at home by a physical therapist, is an evidence-based intervention proven to reduce falls and fall-related injuries in older adults. Despite its effectiveness, widespread adoption has been limited by the personnel and resource demands required for program delivery. Exploring alternative delivery methods, including remote platforms and community-based programs, may help overcome implementation barriers and expand access to this program.

**Objective:**

This study aims to evaluate (1) the effectiveness of 2 delivery modes of the OEP, a remotely delivered, smartphone-based program, and an in-person, community-based program, and (2) participant experiences, feasibility, and acceptability across both delivery formats.

**Methods:**

Thirty-six adults aged 55 years or older, classified as being at intermediate or high risk for falls according to the World Falls Guidelines, will be enrolled in this study. Eligible participants will be randomly assigned to one of two intervention groups: (1) a remotely delivered, smartphone-based OEP, or (2) an in-person, community-based OEP. Participants in the community-based group will attend sessions at a senior center, whereas those in the smartphone-based group will complete the program at home via the Improve (Vipul Lugade) smartphone app. Both groups will receive individualized 60-minute training sessions twice a week for 8 weeks, delivered by certified trainers under the supervision of physical therapists. The 16-session program will include progressive balance, mobility, and strengthening exercises consistent with the OEP. The primary outcome is self-selected gait speed. Secondary outcomes include injurious falls, fall risk assessed using the Physiological Profile Assessment, Timed Up and Go, 30-Second Chair Stand, 4-Stage Balance Test, and 4-Square Step Test, as well as measures of feasibility, acceptability, usability, and health-related quality of life. Outcomes will be assessed at baseline and postintervention by investigators blinded to group allocation. In addition, gait speed will be assessed weekly throughout the 8-week intervention using the Improve app to monitor changes over time. Trainers will be blinded to outcome data, including baseline and interim assessment results.

**Results:**

The study received funding in February 2026, and enrollment began in the same month. As of May 2026, 16 participants have been enrolled. Data collection is expected to be completed in December 2026, with findings anticipated for dissemination by the middle of 2027.

**Conclusions:**

This is the first study to use a smartphone to deliver OEP and to directly compare remote- and community-based delivery platforms. Findings may support broader adoption and implementation of this evidence-based fall prevention program by reducing participation barriers and expanding access for older adults, particularly in remote and underserved areas.

## Introduction

Falls among older adults are a major public health concern, with approximately one in 4 individuals experiencing at least one fall each year [1]. Among those who fall, nearly two-thirds will experience a recurrent fall [2]. Falls are the leading cause of injury-related death in this population [3] and account for nearly 3 million emergency department visits annually [4]. Nonfatal falls contribute approximately US $80 billion in health care costs [5]. Falls may lead to reduced mobility [6], loss of independence [7,8], increased likelihood of long-term care placement [9], and a higher risk of premature mortality [10], all of which impose a substantial burden on older adults, caregivers, and health care systems. As the aging population continues to grow, the burden of falls is expected to increase, making fall prevention an increasingly urgent public health priority.

Fall prevention programs that target balance, strength, and mobility are among the most effective strategies for reducing fall risk in community-dwelling older adults [11-13]. The Otago Exercise Program (OEP) is one of the few evidence-based fall prevention programs specifically designed to improve balance and lower-extremity strength [14-16]. Originally developed in New Zealand, the program is traditionally delivered in the home by a physical therapist over the course of one year. Participation in the OEP has been shown to reduce the incidence of falls and fall-related injuries by approximately 35% among older adults [17]. Although highly effective, the home-based delivery of the program over a prolonged period requires substantial resources and personnel, creating barriers to broader adoption and implementation [18,19]. Alternative delivery systems using digital technologies and community-based implementation with shorter intervention durations may help overcome these challenges.

In recent years, smartphones have become an integral part of daily life, with approximately 90% of older adults in the United States reporting smartphone use in 2025 [20]. The use of smartphones to improve health outcomes is increasing because of the devices’ portability, versatility, and built-in sensors, such as accelerometers, gyroscopes, and cameras. Previous studies have demonstrated that smartphone-based assessments can provide valid and reliable gait information across different environments (eg, indoor and outdoor) [21-24], phone models (eg, various brands and versions) [22-24], and placements (eg, on the body, in a bag, on a belt, or in a pants pocket) [23,25]. In addition, smartphones have been shown to safely deliver balance interventions for older adults [26-28], while also enhancing enjoyment, engagement, and adherence to the exercise programs [27].

Thus, smartphone-based platforms offer a promising alternative to traditional home-based delivery of the OEP. By leveraging digital technologies, interventions can reach older adults who may face barriers to in-person sessions, including geographic distance, limited transportation, shortages of trained personnel, or constrained financial resources. Recent studies have explored digital adaptations of the OEP, including virtual platforms using motion-sensing technologies (eg, Kinect and augmented reality) [29,30], real-time interactions via videoconferencing platforms (eg, Zoom and WeChat) [31,32], and community-based programs delivered via pre-recorded digital video discs [33]. However, the evidence remains limited because of the small number of studies [34], lack of stratification by fall risk [29,33,35], nonrandom group assignment [19], and absence of control groups or reliance on usual care as a control [29,31,33]. Consequently, conclusions regarding the effectiveness of these approaches in improving balance and muscle strength and reducing fall risk are still inconclusive.

Alongside digital platforms, community-based delivery may also be a beneficial approach to promote adoption of the OEP, particularly in settings with limited trained personnel and financial resources. Shubert et al [19] conducted a translational research study comparing 2 implementation models for the OEP: the US model delivered by a physical therapist or physical therapist assistant in the home or outpatient setting and a community-based model (eg, adult foster homes, assisted living facilities) delivered by non–physical therapists under the supervision of a consulting physical therapist. The findings indicated no significant differences in improvements in physical performance at 8 weeks between the 2 models. Despite these findings, several limitations exist. These include the lack of random assignment and limited control over program implementation and data collection procedures [19].

To date, the effectiveness, feasibility, and acceptability of the smartphone-based and community-based delivery of the OEP remain largely unexplored. Therefore, this study aims to address this gap by evaluating the impact of a remotely delivered, smartphone-based OEP compared with an in-person, community-based OEP in terms of fall risk and physical performance and assessing participant experiences and feasibility across both delivery formats. Consistent with recommendations from the World Falls Guidelines Task Force, which provide comprehensive guidance based on individual fall risk stratification, this study will include community-dwelling older adults classified as being at intermediate or high risk for falls [13]. We hypothesize that both delivery formats will improve gait speed, balance, and lower-extremity strength, thereby reducing fall risk among community-dwelling older adults after the 8-week intervention. We further hypothesize that both delivery formats will be perceived as feasible and enjoyable by participants.

## Methods

### Study Design

This study is a parallel-group randomized controlled trial that will be conducted between 2026 and 2027. Thirty-six community-dwelling adults will be randomly assigned to either a remotely delivered, smartphone-based OEP program or an in-person, community-based OEP program. This protocol was developed in accordance with the SPIRIT (Standard Protocol Items: Recommendations for Interventional Trials) guidelines (Checklist 1) and has been registered at ClinicalTrials.gov (NCT07433153).

### Participants and Recruitment

The sample size was calculated based on gait speed, the primary outcome measure used in previous training studies [27,36]. With a power of 0.80, an effect size of 0.27, and an alpha level of 0.05, the estimated sample size required for 2 intervention groups and 2 assessment points is 30 participants, as calculated using G*Power (version 3.1.9.7; Heinrich Heine University Düsseldorf) [37]. To account for potential attrition, we will attempt to recruit 36 community-dwelling adults aged 55 years or older into this study, with 18 adults assigned to each group. Adults aged 55 years or older are included, rather than the typical threshold of 65 years, in order to proactively identify and address fall risk prior to older adulthood. Participants will be recruited through flyers and advertisements at local senior centers, libraries, and community centers; the senior newsletter, in collaboration with the local Office for Aging; and email advertisements sent to older adults previously recruited for a parallel fall risk assessment study. Initial eligibility screening will be conducted over the phone (Figure 1).

Adults will be eligible if they can ambulate at least 10 meters with or without an assistive device, have access to an Android or iOS smartphone, are cognitively intact, based on a score of 18 or higher out of 22 on the Montreal Cognitive Assessment-Blind (MoCA-Blind), can communicate in English, and are categorized as either intermediate or high risk of falling based on the World Guidelines for Falls Prevention and Management for Older Adults [13]. Participants will be classified as being at high risk if they meet any one of the specified criteria. Specifically, these criteria include (1) 2 or more falls in the previous year; (2) a fall resulting in an injury severe enough to require consultation with a physician; (3) a fall resulting in the participant being unable to get up from the floor; (4) loss of consciousness or suspected syncope associated with a fall; or (5) frailty. Thus, meeting any one of these criteria is sufficient for classification as high risk for falls. Participants who do not meet the high-risk criteria will be classified as being at intermediate risk of falling if they have a gait speed ≤0.8 m/s or a Timed Up and Go (TUG) time >15 seconds. Therefore, participants who do not meet the high-risk criteria and have a gait speed >0.8 m/s or a TUG time ≤15 seconds will be classified as being at low risk and will not be eligible for the study. Adults will be excluded if they report a lower-limb amputation, visual impairment that cannot be corrected with lenses, persistent dizziness or lightheadedness, uncontrolled hypertension, diabetes, a neurological or musculoskeletal impairment, pain greater than 7 on a scale of 0‐10, or any spinal or lower-extremity surgery in the past 12 months.

During the phone screening, adults will be provided with a description of the study design and asked yes-or-no questions related to the inclusion and exclusion criteria. To evaluate fall risk, adults will first be provided with a definition of a fall, which is “an unexpected event in which you have come to rest on the ground, floor, or lower level” [13]. They will then be asked to provide (1) the number of falls in the past year, (2) whether any fall resulted in an injury that required a visit to a health care professional, (3) whether they had experienced a fall that left them unable to get up without assistance, and (4) whether they had experienced a fall associated with loss of consciousness or suspected syncope. In addition, the FRAIL Scale will be used to screen for frailty [38]. The Fatigue, Resistance, Ambulation, Illnesses, and Loss of weight (FRAIL) scale consists of 5 questions assessing fatigue, resistance, ambulation, illnesses, and weight loss, with a total score ranging from 0 to 5. Participants who score 1 or higher will be eligible for inclusion in the study and will subsequently undergo cognitive screening using the MoCA-Blind. Those who meet all eligibility criteria and indicate a willingness to participate in the 8-week training program will be scheduled for a baseline assessment at the Motion Analysis Research Laboratory, conducted by investigators not involved in either intervention arm. All screening data from individuals who are deemed ineligible or who decline participation will be securely destroyed.

During the initial visit, participant demographic and clinical characteristics will be obtained. Participants will complete questionnaires regarding their smartphone usage, current exercise and activity level, and medication history. Handgrip strength will be measured on the dominant hand with the participant seated and the elbow kept at a 90-degree angle, in accordance with the clinical assessment recommendations from the American Society of Hand Therapists [39]. In addition, participants will be assessed using the short form of the Geriatric Depression Scale (GDS) and the Falls Efficacy Scale-International (FES-I). The GDS is a validated screening tool for depression in cognitively intact adults and has demonstrated good reliability and validity [40]. The FES-I evaluates fear of falling with strong psychometric properties [41]. The GDS and FES-I were selected based on the World Guidelines for Falls Prevention and Management for Older Adults [13], which identify depressive disorders and fear of falling as factors associated with falls and recommend the GDS and FES-I as potential measurement instruments for multifactorial fall-risk assessment.

**Figure 1.**
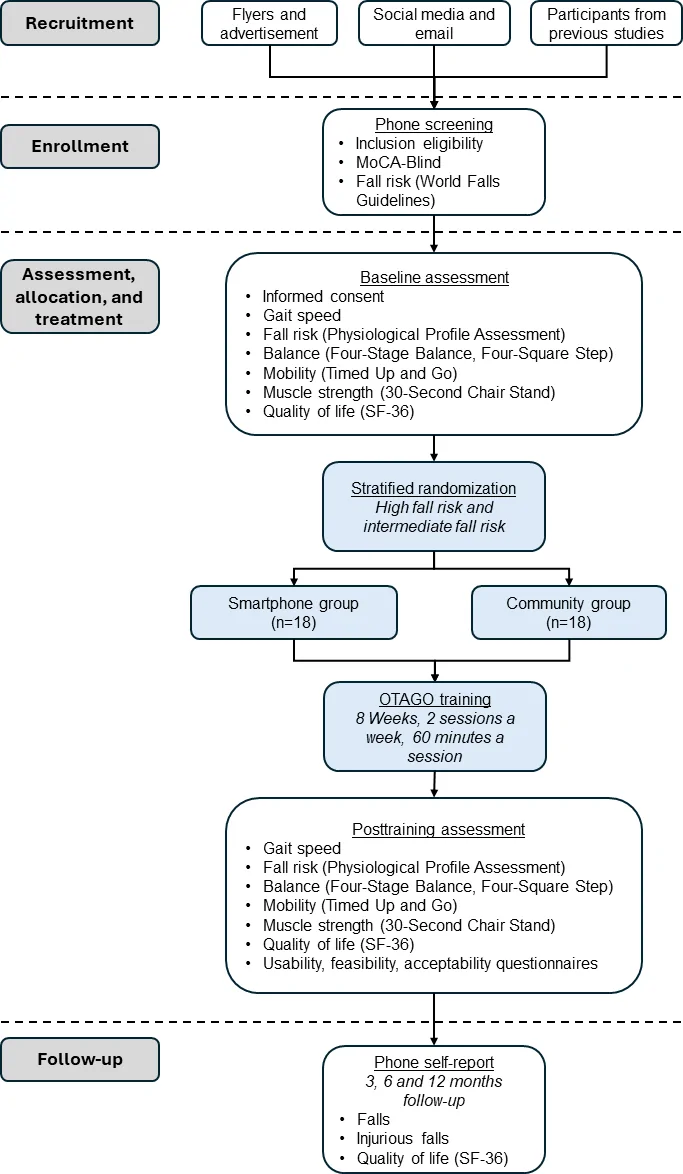
Schematic diagram and timeline of the parallel-group randomized controlled trial comparing a remotely delivered, smartphone-based Otago Exercise Program (OEP) with an in-person, community-based OEP among community-dwelling older adults. The study includes participant recruitment and enrollment, baseline assessment, intervention, and follow-up assessment. MoCA: Montreal Cognitive Assessment; SF-36: 36-item Short Form Health Survey.

### Outcome Measures

All primary and secondary outcome measures will be assessed at 2 time points: approximately one week before the intervention (baseline) and one week after completion of the 8-week training program (posttest). In addition, gait speed will be measured weekly throughout the 8-week intervention using a smartphone equipped with a custom-built Improve app (Vipul Lugade) [25]. At the 3-, 6-, and 12-month follow-up assessments, data on the number of falls, injurious falls, and health-related quality of life, as measured by the 36-item Short Form Health Survey (SF-36), will be collected. Participants will be informed at enrollment about the planned 3-, 6-, and 12-month follow-up assessments and that the study team will contact them at each follow-up time point. Efforts will be made to maintain contact with participants who withdraw from the study and to obtain follow-up outcome data when feasible.

### Primary Outcome

Gait speed was selected as the primary outcome because it is a valid, reliable, and clinically meaningful measure of functional mobility in community-dwelling older adults [42,43]. Gait speed has also been widely described as a “functional vital sign” because it provides a simple and objective measure of overall functional status [44,45] and has been associated with important health outcomes, including disability, hospitalization, and mortality [44-46]. In addition, previous studies have reported improvements in gait speed following OEP interventions in older adults [47,48], supporting the use of gait speed as an objective measure of functional mobility in the present study.

Gait speed will be measured using the 10-meter walk test (10mWT) [49]. Participants will perform 2 trials at a self-selected comfortable gait speed along a level 10-meter walkway. An investigator will record the time required to transverse the middle 6-meter section using a stopwatch, allowing 2 meters for acceleration and 2 meters for deceleration. The 2 trials will be performed consecutively, with rest provided as needed between trials. In addition to the baseline and posttest assessments, self-selected comfortable gait speed will be assessed weekly using the Improve app [25]. The app will record triaxial accelerations and angular velocity and calculate gait speed throughout the trial. The validity and reliability of gait speed derived from the Improve app have been demonstrated across different smartphone placement locations and testing environments in previous research [21,23,25]. Specifically, Improve app-derived gait speed showed strong correlations with measurements from an instrumented walkway when the smartphone was placed on the body (*r*=0.96) and in a pants pocket (*r*=0.90), with excellent test–retest reliability for both placements (intraclass correlation coefficient [ICC]=0.97 and 0.94, respectively) [25]. In addition, gait speed derived from the Improve app has demonstrated strong correlations with motion capture in controlled laboratory environments (*r*=0.91) and with video-based measurements in free-living conditions (*r*=0.83) [23], with excellent test–retest reliability in both laboratory (ICC=0.97) and free-living environments (ICC=0.90) [21].

To ensure consistency within each intervention group, smartphone placement will be standardized within each assessment setting. Participants in the smartphone group will be instructed to carry the smartphone in their pants pocket during the weekly home-based gait assessments, whereas participants in the community group will complete the assessment at the senior center with the smartphone secured to their pants near the lumbar spine. Although smartphone placement and the assessment environment will differ between groups, the same placement will be maintained within each group throughout the study. In both groups, participants will perform 2 trials at a self-selected comfortable gait speed along a level 10-meter walkway. The Improve app will be started at the beginning of the walk and stopped at the end of the 10-meter walkway. Data from the middle 6 meters of the walkway will be extracted for analysis.

### Secondary Outcomes

Information regarding falls and fall-related injuries will be self-reported by participants. Injurious falls or fall-related injuries are defined by the World Falls Guidelines [13] as injuries resulting in medical attention, including hospitalization, following a fall. Injuries include fractures, joint dislocation, head injury, sprain or strain, bruising, swelling, laceration, or other serious injury. In addition, participants will complete the SF-36, which assesses health-related quality of life across 8 health domains: physical functioning, social functioning, role limitations due to physical health, bodily pain, mental health, role limitations due to emotional problems, vitality, and general health perceptions [50].

Fall risk will be assessed using the short form of the Physiological Profile Assessment (PPA) [51]. The PPA evaluates 5 validated measures of physiological function: visual contrast sensitivity (decibels), lower-limb proprioception (degrees of separation), quadriceps muscle strength (kilograms), hand reaction time (milliseconds), and postural sway area (mm²). The PPA composite score will be calculated from these 5 physiological measures and used as an overall measure of physiological fall risk [51]. In addition, quadriceps muscle strength and postural sway will be examined as individual secondary outcome measures to evaluate changes in physiological domains specifically related to lower-extremity strength and postural control, respectively.

For the contrast sensitivity test, the Melbourne Edge Test chart will be positioned at approximately 45 degree. This test assesses participants’ ability to detect edges under low-contrast conditions. The chart contains 20 circles, each bisected by a line oriented vertically, horizontally, or diagonally (to the right or left), with progressively decreasing contrast across the chart. The circles are arranged in 4 rows, with contrast decreasing from the upper left to the lower right. Participants will begin with the circles in the first row and proceed sequentially through rows 2, 3, and 4, from left to right. Participants will view each circle individually and indicate the orientation of the line by pointing to the corresponding match on the response card. The last circle correctly identified will be recorded, and the contrast sensitivity value corresponding to that circle will be used for further analysis. Lower-limb proprioception will be evaluated using a limb-matching task, in which participants will raise both legs simultaneously and attempt to align the position of their great toes with a Perspex sheet placed between the legs. Participants will complete 3 practice trials with their eyes open, followed by 5 test trials with their eyes closed. The mean performance across the 5 test trials will be used for analysis. Quadriceps muscle strength will be assessed using a dynamometer. Participants will be instructed to push with their dominant leg against a strap attached to the dynamometer, which is secured to a horizontal bar at the back of a tall testing chair. Participants will complete 3 trials, and the maximum value will be used for analysis. Rest will be provided between trials as needed. For the hand reaction time task, participants will be instructed to press a mouse button as quickly as possible upon seeing a red light on the mouse, using their dominant hand. Participants will complete 5 practice trials, followed by 10 test trials. The mean reaction time across the 10 testing trials will be used for analysis. Postural sway will be measured with a swaymeter rod secured at the waist while participants stand as still as possible for 30 seconds. One trial will be performed under each of 4 conditions: standing on a firm surface with eyes open, standing on a firm surface with eyes closed, standing on a foam surface with eyes open, and standing on a foam surface with eyes closed. Sway area, calculated as the product of anteroposterior and mediolateral displacement, will be used for analysis.

To assess mobility, participants will complete the TUG test [52]. In 2 trials, participants will be asked to rise from a chair, walk 3 meters, turn around, walk back to the chair, and sit down. Participants will walk at their self-selected comfortable speed, with or without the use of an assistive device. The total time to complete the task will be recorded using a stopwatch. To evaluate lower-extremity muscle strength and endurance, participants will complete one trial of the 30-second chair stand test [53]. The total number of chair rises performed within the 30-second period will be recorded. Adequate rest will be provided following the test before proceeding to the next assessment.

Standing balance will be assessed using the 4-Stage Balance Test [54]. Participants will perform 4 conditions in sequence: standing with the feet together, standing in a semitandem stance, standing in a full tandem stance, and standing on one leg. The time each position is maintained will be recorded using a stopwatch, with a maximum of 10 seconds for each condition. One trial will be performed, with adequate rest provided between conditions as needed. To assess dynamic balance and coordination during multidirectional stepping, participants will complete the Four Square Step Test (FSST) [55]. The test requires participants to step sequentially into 4 squares formed by 4 PVC pipes placed on the floor. Square 1 will be positioned at the upper left, Square 2 at the upper right, Square 3 at the lower right, and Square 4 at the lower left. Participants will begin by standing in Square 1. Timing will start when the first foot contacts Square 2 and stop when the second foot contacts Square 1. Participants will complete one practice trial followed by 2 testing trials. During each trial, the total time required to complete the sequence in both clockwise and counterclockwise directions will be recorded using a stopwatch.

At the end of the intervention program, all participants will complete a questionnaire assessing the acceptability [56], feasibility [57], and usability [58] of their assigned 8-week training regimen. The questionnaire includes Likert-scale items rated from 1 to 5, where 1 indicates “strongly disagree” and 5 indicates “strongly agree.” Example items include: “I enjoyed participating in this exercise program,” “I was able to participate in the program as planned,” and “I thought the system was easy to use,” which assess acceptability, feasibility, and usability, respectively. For participants in the smartphone-based group, “the system” refers to the smartphone app and its exercise instructions, whereas for participants in the community-based group, “the system” refers to the exercise program and the instructions provided during the in-person sessions. Thus, the item is intended to assess the ease of use of the intervention format received by each group. Open-ended questions are also included, such as “What parts of the program were easy or enjoyable?” “What challenges or difficulties did you experience?” and “What changes would improve the program for you?”

### Procedures

#### Randomization and Blinding

Participants will be randomly assigned to one of 2 training groups, with stratification by fall risk level (intermediate or high) based on the World Falls Guidelines classification. Allocation will be performed in a 1:1 ratio by an investigator (VL) who is not involved in testing or training, using a computerized random number generator. All outcome assessments will be conducted by trained Doctor of Physical Therapy (DPT) students who are blinded to the group allocation. Assessors will not be informed of participants’ group assignments and will not attend any intervention sessions. All training will be delivered by a separate group of DPT students under the supervision of consulting physical therapists (PS, WNB, and KG). All trainers are certified in the OEP through the Carolina Geriatric Workforce Enhancement Program at the University of North Carolina at Chapel Hill. Trainers will be blinded to participants’ assessment results and will not have access to baseline or interim assessment data. Because of the nature of the interventions, participants will be aware of both intervention arms and will know their group assignment following randomization after baseline testing.

#### Intervention Arms

All intervention exercises are based on the OEP [15] and will be delivered using two different formats: (1) a remotely delivered, smartphone-based program and (2) an in-person, community-based program. In the smartphone group, the intervention will be delivered through the Improve app (Figure 2). This app, which will be installed on either the participant’s iOS or Android smartphone, will allow participants to review workout instructions and track their prescribed exercises. Instructions will include media in both video and audio formats as well as through written text. The program for the smartphone group will be delivered by 4 OEP-certified DPT student trainers under the supervision of a licensed physical therapist (PS).

**Figure 2.**
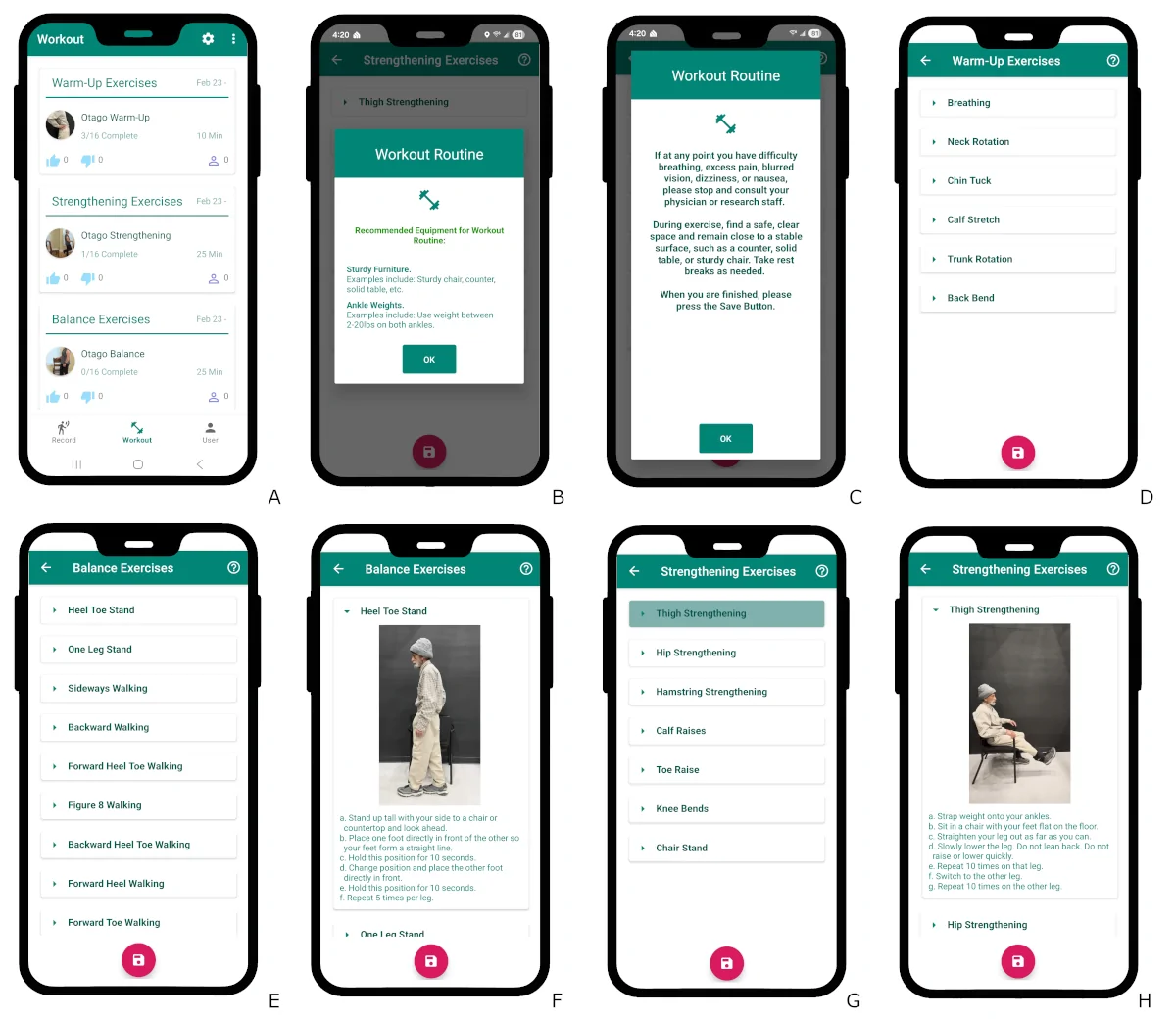
Examples of “Improve” app screens used to remotely deliver the Otago Exercise Program (OEP) to community-dwelling older adults in the smartphone-based intervention group. Screens included: (A) overview of workout domains; (B) recommended equipment; (C) safety guidelines; (D) list of warm-up exercises; (E) list of balance exercises; (F) individual balance exercises presented via video, audio, and text instructions; (G) list of strengthening exercises; (H) individual strengthening exercises presented via video, audio, and text instructions.

All participants in the smartphone group will be provided with an onboarding session prior to initiating the program (Figure 3). This session will include (1) an overview of the study, including goals, schedule, and expectations; (2) review of program rules and safety guidelines; (3) assessment of readiness to begin the program; (4) training on use of the smartphone app; (5) individualized prescription of the OEP based on physical capacity and health status; (6) instruction and demonstration of all exercises, with participants required to demonstrate proper technique and safe performance; (7) education on the safe use of ankle weights; and (8) evaluation of participant readiness, including their ability to perform exercises safely and independently (with assistance if needed), understand progression criteria, identify a safe location within the home for exercise, and effectively use the smartphone app. Participants will be instructed to perform approximately 60 minutes of exercise per session via the Improve app. They will also be asked to record exercise adherence using Otago exercise calendars and diaries, maintain their usual activity patterns, and refrain from initiating new structured physical activity programs during the 8-week study period. Convenient times for regular check-in calls with the trainers will also be established. Participants in the smartphone group will be contacted twice weekly by phone to receive individualized progression guidance, to ensure safety and adherence, and to assist with any troubleshooting of technology-related issues. The twice-weekly telephone contact was designed to provide a comparable frequency of participant–trainer contact to that experienced by participants in the community-based group, who will attend trainer-led sessions twice a week.

The second arm, the in-person, community-based group, will receive identical interventions delivered during group sessions hosted at the Johnson City Senior Center (Figure 4). Groups will be maintained at a size of 8‐12 older adults, with 4 OEP-certified DPT student trainers supervised by a licensed physical therapist (WNB or KG). The community groups will meet at dedicated times twice per week. The onboarding session, conducted during the first visit, will cover the same content and materials as the smartphone group, with the exception of instruction on smartphone use. Participants in this group will not be provided with access to the Improve smartphone app until completion of the study.

**Figure 3.**
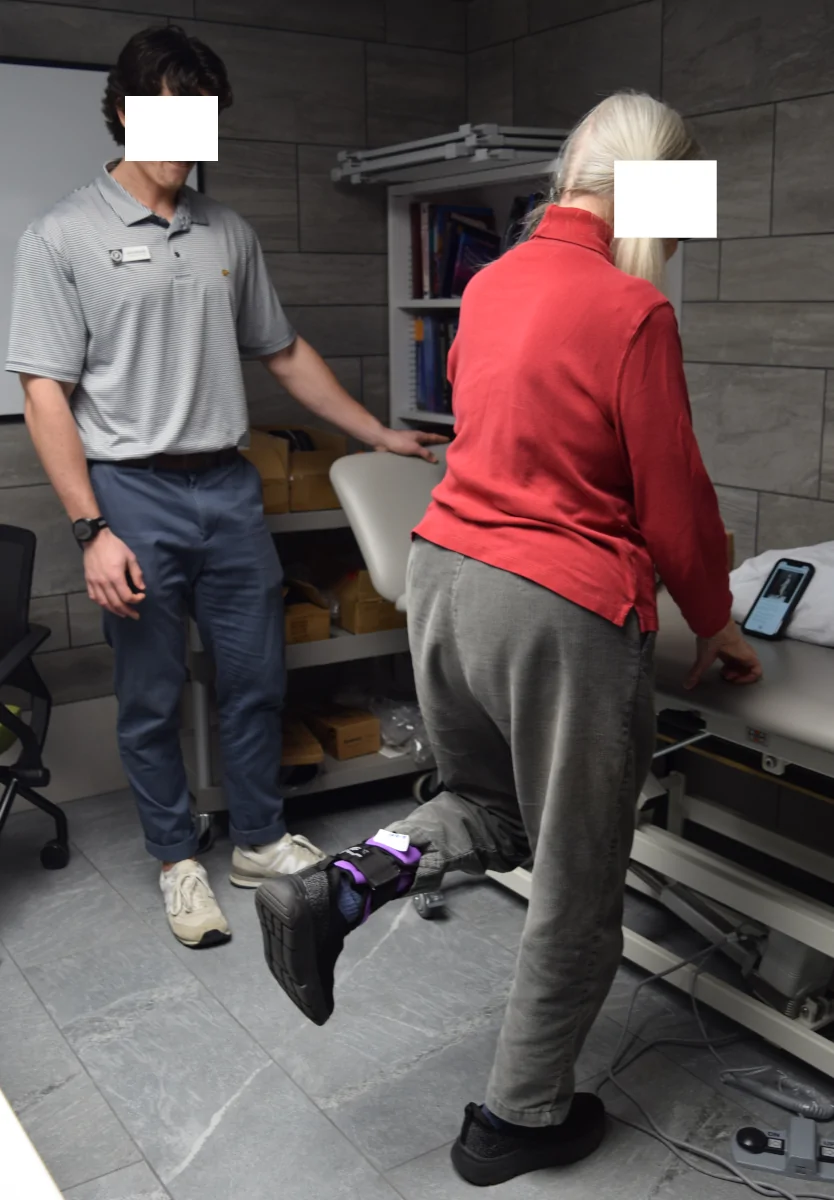
A community-dwelling older adult participating in an onboarding session for the remotely delivered, smartphone-based Otago Exercise Program (OEP) intervention.

**Figure 4.**
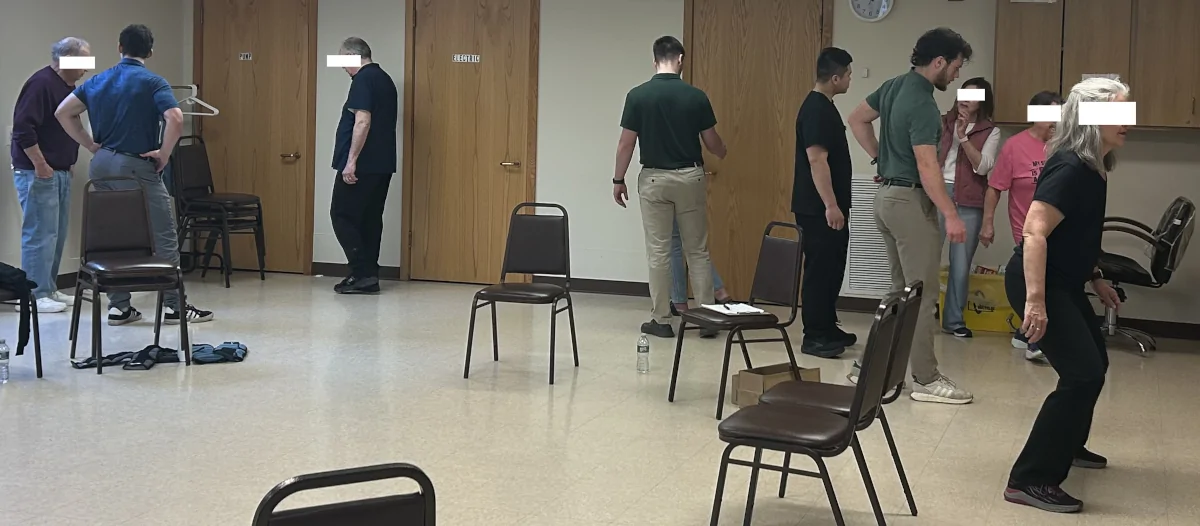
Community-dwelling older adults participating in a supervised, in-person Otago Exercise Program (OEP) session at a senior center, as part of the community-based intervention group.

#### Intervention Program

All participants will perform the same scope of exercises, with progression tailored individually by OEP-certified DPT students. The training program consists of 16 sessions, each lasting approximately 60 minutes, delivered twice weekly for 8 weeks. Across the 16 sessions, participants will complete balance and strengthening activities based on the OEP (Table 1). Consistent with the original OEP, participants will also receive walking guidance and be instructed to complete at least 30 minutes of walking twice per week independently outside of the scheduled exercise sessions. The walking component is separate from and in addition to the balance and strengthening exercises performed during the 60-minute exercise sessions. The 30 minutes of walking may be completed as one continuous session or divided into shorter periods (ie, 3 10-min walks) throughout the day. Walking guidance will include recommendations to wear appropriate footwear and corrective eyewear as needed; use a mobility aid when appropriate; avoid walking outdoors in excessively hot or cold weather; scan the walking path ahead and maintain attention to walking; avoid multitasking while walking; and avoid roads, sidewalks, or other walking surfaces that pose tripping hazards. For indoor walking, participants will be advised to remove or avoid potential hazards, including scatter rugs, loose carpeting, electrical cords, and uneven flooring, and to be aware of pets or other obstacles in the walking environment.

The exercise dose for the balance and strengthening components was adapted from the original OEP protocol to accommodate the feasibility and accessibility of community-based delivery. The original OEP recommends 3 sessions of 30 minutes per week of strength and balance exercises (90 minutes per week). However, delivering 3 in-person sessions per week was not feasible within the scheduling constraints of the participating senior center and would have increased the participation burden for older adults. Because the participating senior center could accommodate only 2 in-person sessions per week, the duration of each exercise session was increased to 60 minutes to compensate for the reduced frequency of exercise sessions, resulting in 120 minutes of prescribed strengthening and balance exercise per week. Accordingly, both the smartphone-based and community-based groups will complete the balance and strengthening component twice weekly for 60 minutes per session for 8 weeks. This adaptation was informed by previous research demonstrating the effectiveness of group-based OEP delivered twice weekly [59]. Although the adapted schedule differs from the original OEP dose, it was designed to retain the strength and balance exercises prescribed by the OEP while facilitating feasible implementation in a community-based setting. The 8-week intervention duration was selected based on the original OEP protocol, which includes an 8-week physical therapist management phase, as well as previous studies demonstrating the effectiveness of home-based and virtually delivered OEP interventions over 8 weeks [19,29,60].

For all exercises, participants will be instructed to breathe naturally while performing each exercise. For each flexibility activity, participants will be asked to slowly stretch to the end ranges of motion while ensuring they do not push into pain. For balance exercises, participants are asked to perform movements near a counter, sturdy chair, or wall for support. For strengthening activities, participants are instructed to use only the provided adjustable ankle weights as prescribed to reduce the risk of injury. Participants will also be instructed to perform each strengthening exercise slowly, taking approximately 2‐3 seconds to lift the weight during the concentric phase and 4‐5 seconds to lower the weight during the eccentric phase. For both intervention groups, the recommended exercise tempo will be taught and practiced during the onboarding session and reinforced throughout the intervention period. For participants in the smartphone group, adherence to the recommended exercise tempo will be discussed and reinforced during the twice-weekly telephone calls. Participants will be instructed to rest for 1‐2 minutes between sets.

For each exercise routine, the modified Borg rate of perceived exertion (RPE) [61] or the Talk Test [62] will be used to monitor the intensity of exercise activities. The rate of perceived stability (RPS) [63] will be used to monitor the difficulty of balance activities. The RPE and RPS are scored on scales of 1‐10, where lower numbers represent reduced effort and higher numbers represent maximal effort.

**Table 1.** Description of exercises provided during the 8-week intervention to older adults participating in the in-person, community-based OEP^a^ and remotely delivered, smartphone-based OEP.

Category Exercise		Description
Warm-up		
Breathing		Stand tall, breathe deeply through the nose, and exhale gently through the mouth
Neck rotation		Slowly turn the head as far as possible to the left and right without pain
Chin tuck		Gently glide the head straight back as if making a “double chin”
Calf stretch		While seated, extend the leg and dorsiflex the ankle
Trunk rotation		Stand tall with hands on hips and rotate the trunk as far as comfortably possible to the left and right
Back bend		Stand tall and gently extend the trunk by pressing the hips forward and lifting the chest toward the ceiling
Strengthening		
Knee extension		Sit in a chair with feet flat on the floor. Slowly extend and lower each leg
Hip abduction		Stand tall and perform straight-leg abduction with each leg
Hamstring curls		Stand tall and flex the knee, bringing the heel toward the buttocks
Calf raises		Stand and perform plantarflexion by lifting the heels off the ground
Toe raises		Stand and perform dorsiflexion by lifting the forefoot off the ground
Sit-to-stand		Sit in a chair and rise to a standing position
Knee bends		Perform squats, with depth determined by when heels start to lift from the floor
Balance		
Heel-toe stand		Tandem stance, alternating which leg is placed in front
One-leg stand		One leg stance, alternating legs
Sideways walk		Repeat 10 steps sideways walking in both directions
Backward walk		Perform backward walking
Forward heel toe walk		Perform tandem forward walking
Figure 8 walk		Walk in a figure of 8 pattern, first in the clockwise direction, then in the counterclockwise direction
Backward heel toe walk		Perform tandem backward walking
Forward heel walk		Perform forward walking while keeping weight supported on the heels only
Forward toe walk		Perform forward walking while keeping weight supported on the forefoot only
Stair climbing		Walk up and down a set of stairs

a OEP: Otago Exercise Program.

#### Exercise Progression

For strengthening activities, participants will perform strengthening exercises at moderate to high intensity, targeting 7‐8 out of 10 on the RPE scale [61]. A 10 repetition maximum (10 RM) will first be determined by having participants lift an estimated weight through the full range of motion as many times as possible, with a concentric phase of 2‐3 seconds and an eccentric phase of 4‐5 seconds. The 10 RM is reached when muscle fatigue causes loss of proper form and limits full range of motion during the final 1‐2 repetitions of a 10-repetition set.

Once established, participants will perform 2 sets of 10 repetitions at the 10 RM weight. Resistance will be decreased if pain or prolonged delayed-onset muscle soreness occurs. Exercise intensity will be monitored using the RPE or the Talk Test, in which participants should be able to speak in short sentences but not sing, with audible breathing [62]. Resistance will be progressed by increasing the ankle weights when participants complete 2 sets of 10 repetitions with an RPE below 7.

Balance exercises will be progressed from performing movements while holding onto a stable support with both hands initially to 1 hand, then to fingertip support, and eventually without support. Additional progression will be achieved through increasing the number of repetitions and the longer duration of each exercise. Exercise intensity will be monitored using the RPS [63], with a target range of 7‐8, corresponding to the ratings “moderately unbalanced” to “unbalanced.

### Statistical Analysis

The 4 DPT students who will conduct all baseline and posttest assessments completed reliability testing for the TUG, 10mWT, PPA proprioception measure, and FSST. A total of 10 young adults (6 women, aged mean 20.4, SD 0.8 years) completed 2 trials of each of the 4 activities, with average values obtained by each of the 4 raters. An ICC (ICC(2,1)) will be conducted to evaluate interrater reliability [64].

For all outcome measures, normality of data will be evaluated graphically and numerically using the Shapiro-Wilk test prior to statistical tests of significance. Outliers will be examined for potential technical or measurement errors, and those not attributable to such errors will be included in all analyses. Patterns of missing data will be evaluated prior to any analysis. If data are missing at random, appropriate methods such as multiple imputation will be used as appropriate. Sensitivity analyses will compare results from complete-case and imputed datasets with clear procedures elucidated.

Differences in group demographics will be examined using an independent sample *t* test or their nonparametric equivalents. A 2-way repeated-measures ANOVA with the Bonferroni correction will be used for each dependent variable, with mode of delivery (ie, smartphone vs community) as the between-subjects factor and visit (ie, baseline vs posttest) as the within-subjects factor. If baseline differences between groups are identified, an analysis of covariance (ANCOVA) will be performed, with baseline factors included as covariates. Effect sizes will be reported as partial eta squared (*η^2^*), alongside the *F* statistics and *P* values. Differences in self-reported effectiveness, enjoyment, and feasibility of the exercise programs between groups will be investigated using independent-samples Mann-Whitney *U* tests. SPSS (version 28.0; IBM Corp) will be used for all statistical analyses, with alpha levels set at 0.05.

### Ethical Considerations

The study will be conducted in accordance with the Declaration of Helsinki. Written informed consent, as approved by the Binghamton University Institutional Review Board, will be obtained from all participants by a trained investigator (VL) prior to baseline assessment. Institutional review board approval has been obtained for this randomized trial (STUDY00006790). An independent data monitoring committee was deemed unnecessary because of the low-risk nature of the intervention, the short study duration, and the enrollment of participants who are not considered a vulnerable population.

Participants will be made aware of all potential harms when they enroll in this study. Potential harms include risk of stress or anxiety when performing assessments in the laboratory environment. Additional sources of potential stress include participating in a group setting for adults randomized into the community group or when using the smartphone app if randomized into the smartphone group. For all exercises, there is a risk of balance loss, discomfort, muscle soreness, pain, or injury. Finally, participants will be notified that there is a potential risk for breach of confidentiality.

To protect against or minimize potential harms, participants will be provided with adequate education and time to understand the study protocols and risks. Participants will further be made aware of the voluntary nature of any questionnaires, assessments, and prescribed exercises. To reduce harms related to smartphone usage, participants will complete an onboarding session to provide adequate practice in using the smartphone app. Participants will also be encouraged to contact the trainers if they have any questions during the study.

Participants will be made aware that muscle soreness is expected upon starting any new exercise program. To ensure safety, participants will be encouraged to complete all exercises in a clutter-free home environment, with a family member or caregiver nearby. In the community setting, trainers will ensure safe completion of all tasks. For all participants, adequate rest between trials will be encouraged with appropriate clothing and availability of support surfaces such as chairs, walls, and counters. To reduce potential risks from breach of confidentiality, all data will be deidentified, with a unique subject identification code associated with the data. Only the investigators (PS, VL, WNB, and KG) will have access to a list indicating a subject code to identifiable information.

Participants will be asked to report any changes in health status, adverse events, or other concerns that may occur during the study period. For participants in the in-person, community-based group, these changes will be discussed with the trainer during the twice-weekly intervention sessions. For participants in the smartphone group, these changes will be discussed during the twice-weekly telephone calls that are part of the intervention. Thus, these discussions will be incorporated into the scheduled intervention contacts and will not require additional visits or assessments. An adverse event will include any untoward medical occurrence during participation in this clinical study. Adverse events will be labeled according to severity, which is based on their impact on the participant. An adverse event will be termed “mild” if it does not have a major impact on the participant, “moderate” if it causes the participant some minor inconvenience, and “severe” if it causes a substantial disruption to the participant’s well-being. Adverse events will further be categorized according to the likelihood that they are related to the study intervention. Specifically, they will be labeled definitely unrelated, possibly related, probably related, or definitely related to the study intervention. All adverse events will be reported to the Institutional Review Board. This study will be stopped prior to its completion if: (1) the intervention is associated with adverse effects that raise concerns regarding its safety, (2) difficulties in participant recruitment or retention significantly compromise the ability to evaluate the study endpoints, (3) new information becomes available during the trial that necessitates termination, or (4) other circumstances occur that warrant discontinuation of the trial.

## Results

Data collection is ongoing and began following receipt of study funding in February 2026. As of May 2026, 16 participants had enrolled, with 8 assigned to the smartphone group and 8 to the community group (Figure 5). One participant in the smartphone group withdrew for medical reasons. Eight participants had started the OEP at the senior center, and 7 participants had begun the program delivered via the smartphone app. After program initiation, one participant in the community group has discontinued the intervention for medical reasons unrelated to the program. Participant enrollment is expected to be completed by December 2026, and study findings are anticipated to be disseminated by the middle of 2027. Upon completion, all participants will be contacted and provided with a summary of the study findings.

**Figure 5.**
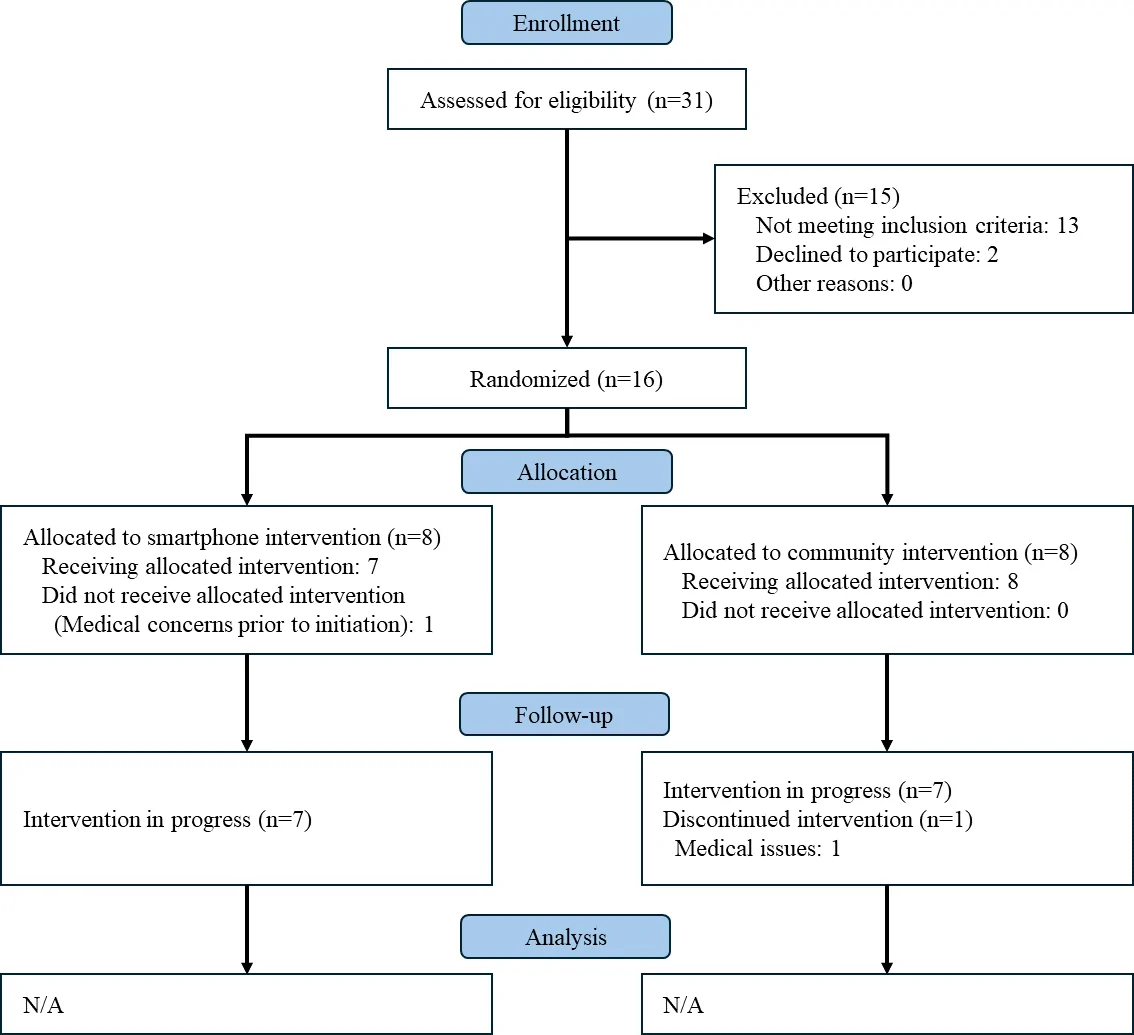
Flow diagram showing participant enrollment, allocation, and follow-up to date in the parallel-group randomized controlled trial comparing a remotely delivered, smartphone-based Otago Exercise Program (OEP) with an in-person, community-based OEP among community-dwelling older adults.

## Discussion

This is the first study to examine the use of a smartphone to deliver the OEP and to compare 2 delivery modes: a remotely delivered, smartphone-based program and an in-person, community-based program. We hypothesize that both delivery methods will be similarly effective in improving gait speed, which is considered the sixth vital sign of functional status and overall health [45]. In addition, participants in both groups are expected to demonstrate comparable reductions in fall risk through improvements in balance, lower-extremity strength, and mobility. Both delivery formats are also anticipated to be perceived as feasible and enjoyable by participants and to enhance their health-related quality of life.

The OEP has previously been shown to be effective in reducing falls and fall-related injuries when delivered by a physical therapist in the home setting over a 1-year period [15-17]. Despite its established effectiveness, adoption of the program remains limited because of resource constraints, particularly the time, personnel, and financial resources required for implementation. To address these barriers, digital and community-based delivery platforms may offer feasible alternatives.

Prior studies have explored a variety of digital health approaches, including augmented reality [30], videoconferencing platforms [31], and prerecorded digital video discs [33] to deliver the OEP in home and community settings. For example, Shubert et al [29] conducted a quasi-experimental study using a single-group pre-post design to examine changes in physical performance outcomes, including the Timed Up and Go, 30-Second Chair Stand, tandem stance, single-leg stance, and Berg Balance Scale, following 8 weeks of OEP delivery using a Kinect camera and computer system. Significant improvements were observed across all outcome measures from baseline to postintervention. However, conclusions regarding the effectiveness of this virtual delivery method remain limited because of the absence of a control group and lack of randomized group assignment. Importantly, to the best of our knowledge, no previous studies have investigated the use of smartphones to deliver the OEP, despite the widespread use, portability, and accessibility of smartphones among older adults. Furthermore, although the World Falls Guidelines Task Force [13], a global multidisciplinary group of experts and stakeholders, recommends individualized approaches and stratified fall prevention strategies based on individual risk, this approach has not been widely implemented.

This randomized controlled trial is designed to address existing gaps in previous OEP studies by using randomized group assignment and an active, comparable intervention group. In addition, this study applies the fall risk stratification recommended by the World Falls Guidelines [13], categorizing participants as being at low, intermediate, or high risk. Only participants classified as being at intermediate or high risk will be recruited, as tailored balance and strengthening exercises are expected to provide the greatest benefit for these groups. Although this study incorporates several methodological strengths, several limitations should be acknowledged. These include a relatively small sample size and the geographic location of the study, which may limit generalizability. Another limitation is that, although relevant outcome measures were selected, laboratory-based assessment tools capable of directly quantifying balance performance (ie, the relationship between the center of mass and base of support), such as a motion analysis system, will not be used [65]. Given that the goal of this study is to promote broader adoption of the OEP in community settings, we prioritized assessments that are practical and feasible in real-world settings.

A further potential limitation is that the weekly gait speed assessments will be conducted under different conditions between groups. Participants in the smartphone group will complete the assessments independently in their home environment with the smartphone placed in a pants pocket, whereas participants in the community group will complete the assessments at the senior center with the smartphone secured near the lumbar spine under tester supervision. Although smartphone-based gait speed assessment has demonstrated validity and reliability across different device placement locations and testing environments [23-25], these contextual differences may introduce variability when comparing longitudinal gait speed measurements between groups. These differences were incorporated to preserve the distinction between the smartphone-based and community-based intervention conditions while minimizing additional smartphone exposure in the community group. Therefore, differences in the assessment environment, smartphone placement, and assessment experience should be considered as a potential limitation when interpreting longitudinal between-group changes in gait speed and participant-reported outcomes.

The study also has a potential limitation related to the randomization procedure. Randomization was performed by a study investigator using a computerized random number generator rather than through a centralized randomization system, such as REDCap (Vanderbilt University) or an independent randomization service. Although the investigator responsible for randomization was not involved in participant testing or intervention delivery, the lack of centralized randomization may introduce a potential for selection bias. Future studies could consider centralized randomization procedures to further strengthen allocation concealment. Finally, participants in the smartphone group will record their exercise adherence and any notes about their exercise sessions in paper-based Otago exercise calendars and diaries rather than in the Improve app. These paper-based tools provide a simple record of exercise participation and allow participants to document their experiences, including difficulties performing an exercise, perceived improvements, and how they felt during or after exercise sessions. However, the use of a paper-based recording method may introduce a difference in the intervention experience compared with an approach in which exercise adherence and participant notes are fully integrated into the smartphone platform. Future studies could consider incorporating exercise adherence monitoring and a space for participant notes directly into the smartphone app to provide a more integrated digital experience.

If the findings demonstrate the effectiveness of both delivery formats, the study will provide strong evidence supporting the implementation of digital health and community-based platforms as strategies to enhance the adoption and dissemination of this evidence-based fall prevention program. Smartphone-based delivery may allow older adults to engage in training at their convenience across various locations and times without the need for travel, thereby potentially improving accessibility and adherence and reducing travel-related costs. Community-based delivery, in contrast, allows multiple participants to train simultaneously and may enhance participant enjoyment, potentially improving adherence while optimizing time and personnel resources. Collectively, these approaches may reduce participation barriers and expand access for older adults, particularly those in remote or underserved areas.

## Supplementary material

10.2196/102920 Checklist 1 SPIRIT checklist.

## Abbreviations

10 RM: 10 repetition maximum
10mWT: 10-meter walk test
ANCOVA: analysis of covariance
DPT: Doctor of Physical Therapy
FES-I: Falls Efficacy Scale-International
FRAIL: Fatigue, Resistance, Ambulation, Illnesses, and Loss of Weight
FSST: Four Square Step Test
GDS: Geriatric Depression Scale
ICC: intraclass correlation coefficient
MoCA-Blind: Montreal Cognitive Assessment-Blind
OEP: Otago Exercise Program
PPA: Physiological Profile Assessment
RPE: rate of perceived exertion
RPS: rate of perceived stability
SF-36: 36-item Short Form Health Survey
SPIRIT: Standard Protocol Items: Recommendations for Interventional Trials
TUG: Timed Up and Go
